# Feasibility study of simultaneous multislice diffusion kurtosis imaging with different acceleration factors in the liver

**DOI:** 10.1186/s12880-021-00661-w

**Published:** 2021-09-09

**Authors:** Hui Xu, Nan Zhang, Da-Wei Yang, Ahong Ren, Hao Ren, Qian Zhang, Jin-Xia Zhu, Gui-Jin Li, Zheng-Han Yang

**Affiliations:** 1 Department of Radiology, Beijing Friendship Hospital, Capital Medical University, 95 Yong an Road, Xicheng District, Beijing, 100050 China; 2 Clinical Epidemiology and EBM Unit, Beijing Friendship Hospital, Capital Medical University, Beijing, 100050 China; 3 MR Collaboration, Siemens Healthineers Ltd., Beijing, China; 4 MR Application, Siemens Healthineers Ltd., Guangzhou, China

**Keywords:** Diffusion kurtosis imaging, Simultaneous multislice, Liver, Magnetic resonance imaging

## Abstract

**Background:**

Simultaneous multislice diffusion-weighted imaging (SMS-DWI) has been used to reduce image acquisition time. The purpose of this study was to investigate the feasibility of diffusion kurtosis imaging (DKI) based on the SMS technique in the liver and the influence of this method compared with that of conventional DWI sequences on image quality and DKI-derived quantitative parameters.

**Methods:**

Forty volunteers underwent SMS-DWI sequences with acceleration factors of 2 and 3 (SMS2-DWI, SMS3-DWI) and conventional DWI (C-DWI) of the liver with three b-values (50, 800, 2000 s/mm^2^) in a 3T system. Qualitative image quality parameters and quantitative measurements of the signal-to-noise ratio (SNR), mean kurtosis (MK), mean apparent diffusivity (MD) and apparent diffusion coefficient (ADC) for the liver were compared between the three sequences.

**Results:**

The scan times of C-DWI, SMS2-DWI, and SMS3-DWI were 4 min 11 s, 2 min 2 s, and 1 min 34 s, respectively. For all image quality parameters, there were no significant differences observed between C-DWI and SMS2-DWI (all *p* > 0.05) in the images with b-values of 800 and 2000 s/mm^2^. C-DWI and SMS2-DWI exhibited better scores than SMS3-DWI (all *p* < 0.01) in the images with b-values of 2000 s/mm^2^. In the images with b-values of 800 s/mm^2^, C-DWI and SMS2-DWI exhibited better scores than SMS3-DWI for artefacts and overall image quality (all *p* < 0.01), and C-DWI exhibited better scores than SMS3-DWI for the visibility of intrahepatic vessels (*p* < 0.001). There were no significant differences in the sharpness of the right lobe edge (*p* = 0.144), conspicuity of the left lobe (*p* = 0.370) or visibility of intrahepatic vessels (*p* = 0.109) between SMS2-DWI and SMS3-DWI. There were no significant differences in the sharpness of the right lobe edge (*p* = 0.066) or conspicuity of the left lobe (*p* = 0.131) between C-DWI and SMS3-DWI. For the b-value of 800 s/mm^2^, there were no statistically significant differences between SMS2-DWI and C-DWI (*p* = 1.000) or between SMS2-DWI and SMS3-DWI (*p* = 0.059), whereas SMS3-DWI had a significantly lower SNR than C-DWI (*p* = 0.024). For the DKI-derived parameters (MK and MD) and ADC values, there were no significant differences between the three sequences (MK, *p* = 0.606; MD, *p* = 0.831; ADC, *p =* 0.264).

**Conclusions:**

SMS-DWI with an acceleration factor of 2 is feasible for the liver, resulting in considerable reductions in scan time while maintaining similar image quality, comparable DKI parameters and ADC values compared with those of C-DWI.

## Background

Diffusion kurtosis imaging (DKI), first proposed by Jensen et al. [1], has been widely used to evaluate the degree of liver fibrosis, tumour characteristics, and treatment responses [2–4]. This imaging method requires ultrahigh b-values (1500–2000 s/mm^2^) to facilitate the successful capture of non-Gaussian behaviour [5, 6]. However, ultrahigh b-value DWI results in a decrease in the signal-to-noise ratio (SNR) and an increase in anatomic distortions. To obtain good image quality, increasing the average time for ultrahigh b-values is essential and leads to relatively long scan times. Therefore, the technique of reducing the scan time is very important for promoting the routine application of DKI sequences for the liver.

Recently, simultaneous multislice DWI (SMS-DWI) has been used to reduce the image acquisition time [7–10]; SMS-DWI is based on the excitation of multiple slices simultaneously and subsequent antialiasing to reconstruct the magnitude images. Slices are separated using information about coil sensitivities from phased array coils, gradients, or radiofrequency encoding. The SMS technique shortens the scan time by increasing the number of acceleration factors (AFs), and it was initially used in the brain [11]. In recent years, several studies have reported that the SMS technique shortens the scan time of DWI in the liver, pancreas, prostate, rectum, breast, and kidney [12–16]. A limited number of studies have focused on intravoxel incoherent motion (IVIM) analysis based on SMS-DWI sequences and found a significant scan time reduction [13, 17]. Phi Van et al. reported slight systematic deviations in the IVIM parameters with increased AFs [17]. However, no studies have examined the impact of DKI parameter calculations based on SMS techniques in the liver.

Therefore, the purpose of our study was to investigate the feasibility of DKI based on the SMS technique in the liver and the influence of this method compared with conventional DWI sequences on image quality and DKI-derived quantitative parameters.

## Methods

### Subjects

A total of 40 volunteers (23 females and 17 males; age range 24–53 years, mean 37 years) without a known history of alcohol or drug abuse, viral hepatitis, or prior liver surgery were enrolled.

### DWI protocols

All DWI data were acquired on a 3.0T whole-body MRI scanner (MAGNETOM Prisma, Siemens Healthcare, Erlangen, Germany). An 18-channel phased-arrayed body coil in combination with 12 elements of a 32-channel spine coil was used.

In all the participants, SMS-DWI sequences with acceleration factors of 2 and 3 (SMS2-DWI and SMS3-DWI, respectively) and conventional DWI (C-DWI) were scanned. DWI was acquired in axial slice orientation with a slice thickness of 6 mm in free breathing without respiratory compensation techniques such as respiratory triggering and navigator triggering. Diffusion gradients were applied in four-scan traces with b-values of 50, 800 and 2000 s/mm^2^. Fat suppression with a spectral attenuated inversion recovery (SPAIR) technique was used. Table 1 provides detailed sequence parameters, including scan time.

**Table 1:** Sequence parameters

Scan Parameters	C-DWI	SMS2-DWI	SMS3-DWI AF3
Echo time, TE (ms)	59	61	61
Repetition time, TR (ms)	5200	2400	1800
FoV read (mm)	420	420	420
FoV phase (%)	65.7	65.7	65.7
In-plane resolution (mm^2^)	1.6 × 1.6	1.6 × 1.6	1.6 × 1.6
Slice thickness (mm)	6	6	6
No. slices	30	30	30
Bandwidth, Hz/px	2666	2488	2488
Fat saturation	SPAIR	SPAIR	SPAIR
Shim mode	Standard	Standard	Standard
iPAT	GRAPPA 2	GRAPPA 2	GRAPPA 2
Slice acceleration factor	None	Factor 2	Factor 3
Diffusion mode	4-scan trace	4-scan trace	4-scan trace
b-values(averages) (s/mm^2^)	50(1), 800(4), 2000(6)	50(1), 800(4), 2000(6)	50(1), 800(4), 2000(6)
Acquisition time, min:s	4:11	2:02	1:34

C-DWI, conventional diffusion-weighted imaging; SMS2-DWI, simultaneous multislice accelerated DWI with an acceleration factor of 2; SMS3-DWI, simultaneous multislice accelerated DWI with an acceleration factor of 3; TR, time of repetition; TE, echo time; BW, bandwidth; iPAT, integrated parallel acquisition technique; GRAPPA, generalized autocalibrating partially parallel acquisition; SPAIR, spectral attenuated inversion recovery

### Image assessment

#### Qualitative analysis of image quality

All the images were transferred to a workstation (Syngo.via, VB10; Siemens Healthcare, Erlangen, Germany).

The quality of the images with b-values of 800 s/mm^2^ and 2000 s/mm^2^ was independently evaluated by two radiologists with 5 and 12 years of experience in liver MRI. The two radiologists were blinded to the sequence information.

A five-point Likert scale was used to rate the image quality with respect to the following aspects (sharpness of the right lobe edge, conspicuity of the left lobe, visibility of intrahepatic vessels, absence of artefacts and overall image quality), with higher scores indicating better image quality (5 = excellent, 4 = good, 3 = moderate, 2 = poor, 1 = not diagnostic). Regarding the occurrence of artefacts, 5 = no artefacts, 4 = mild artefacts, 3 = moderate artefacts, 2 = severe artefacts, and 1 = severe artefacts representing no diagnosis. The image quality scores of conventional DWI, SMS2-DWI and SMS3-DWI sequences assigned by the two radiologists were recorded for each participant.

#### Quantitative analysis of signal-to-noise

The signal-to-noise (SNR) ratio was calculated as the ratio between the average signal intensity of the liver parenchyma and the standard deviation of the signal intensity of the air for images with b-values of 800 and 2000 s/mm^2^ by the following formula:

$${\rm SNR}={\rm SI}_{\rm liver}/{\rm SD}_{\rm background}.$$

where SI_liver_ is the mean signal of the liver parenchyma and SD_background_ is the standard deviation of the background noise on the outside sides of the body in the phase-encoding directions.

#### Quantitative analysis of DKI parameters and ADC values

All the images were postprocessed using prototypic software (Body Diffusion Toolbox; Siemens Healthcare, Erlangen, Germany) to extract DKI parameter and ADC maps.

The apparent mean kurtosis (MK) maps and apparent mean diffusivity (MD) maps were calculated using all 3 b-values according to the following equation:

$${\rm S}({\rm b})/{\rm S}(0)={\rm exp} (-{\rm b}\times{\rm MD}+{\rm b}^{2}\times{\rm MD}^{2}\times{\rm MK}/6).$$

where MK is the mean kurtosis coefficient (a unitless parameter that represents the deviation of water motion from the Gaussian distribution) and MD is a corrected mean ADC without a Gaussian bias.

The ADC maps were calculated using all 3 b-values with the following equation:

$${\rm S}({\rm b})/{\rm S}(0)={\rm exp}(-{\rm b}\times{\rm ADC}).$$

Regions of interest (ROIs) were placed by a radiologist with 12 years of experience in abdominal MRI. ROIs with a diameter between 2 and 4 cm were manually placed in liver parenchyma segment V at the portal hilum level on the b 50 s/mm^2^ images of C-DWI and then copied to the corresponding MK, MD and ADC maps of the C-DWI, SMS2-DWI and SMS3-DWI sequences at the same level and position, making sure to avoid large vessels and severe artefacts. The radiologist performed the first measurement within 2 days after the completion of the DWI examination and then performed a second measurement 3 weeks later. Eventually, the average of all the measurements was adopted for analysis.

### Statistical analysis

Statistical analyses were performed using SPSS version 26.0 software (IBM Corp., Armonk, NY USA) and MedCalc Software version 18.2.1 (MedCalc Software bvba, Mariakerke, Belgium). *P* values of less than 0.05 were considered statistically significant.

Image quality parameters among the three DWI sequences (C-DWI, SMS2-DWI, and SMS3-DWI) were compared using the Friedman test. If the Friedman test showed statistical significance, the Dunn-Bonferroni post hoc test was performed to analyse all pairwise comparisons. The interobserver agreement on image quality was analysed by calculating linear weighted Cohen’s kappa, with kappa values of 0.01–0.20 representing slight agreement, 0.21–0.40 fair, 0.41–0.60 moderate, 0.61–0.80 good, and 0.81–1.00 almost perfect agreement.

The differences in the SNR, DKI-derived quantitative parameters and ADC values between the three sequences were compared using one-way ANOVA, and pairwise Bonferroni post hoc tests were performed if the ANOVA showed a significant difference. The distribution and concordance of DKI parameters and ADC values between the three sequences are shown in a Bland–Altman plot. The mean difference and 95% limits of agreement were reported.

## Results

All the sequences were successfully scanned, and the specific absorption rate (SAR) remained below individual limits in all sequences without the need for switching to the first-level mode.

### Scan time

The acquisition times of C-DWI, SMS2-DWI, and SMS3-DWI were 4 min 11 s, 2 min 2 s, and 1 min 34 s, respectively.

**Fig. 1:**
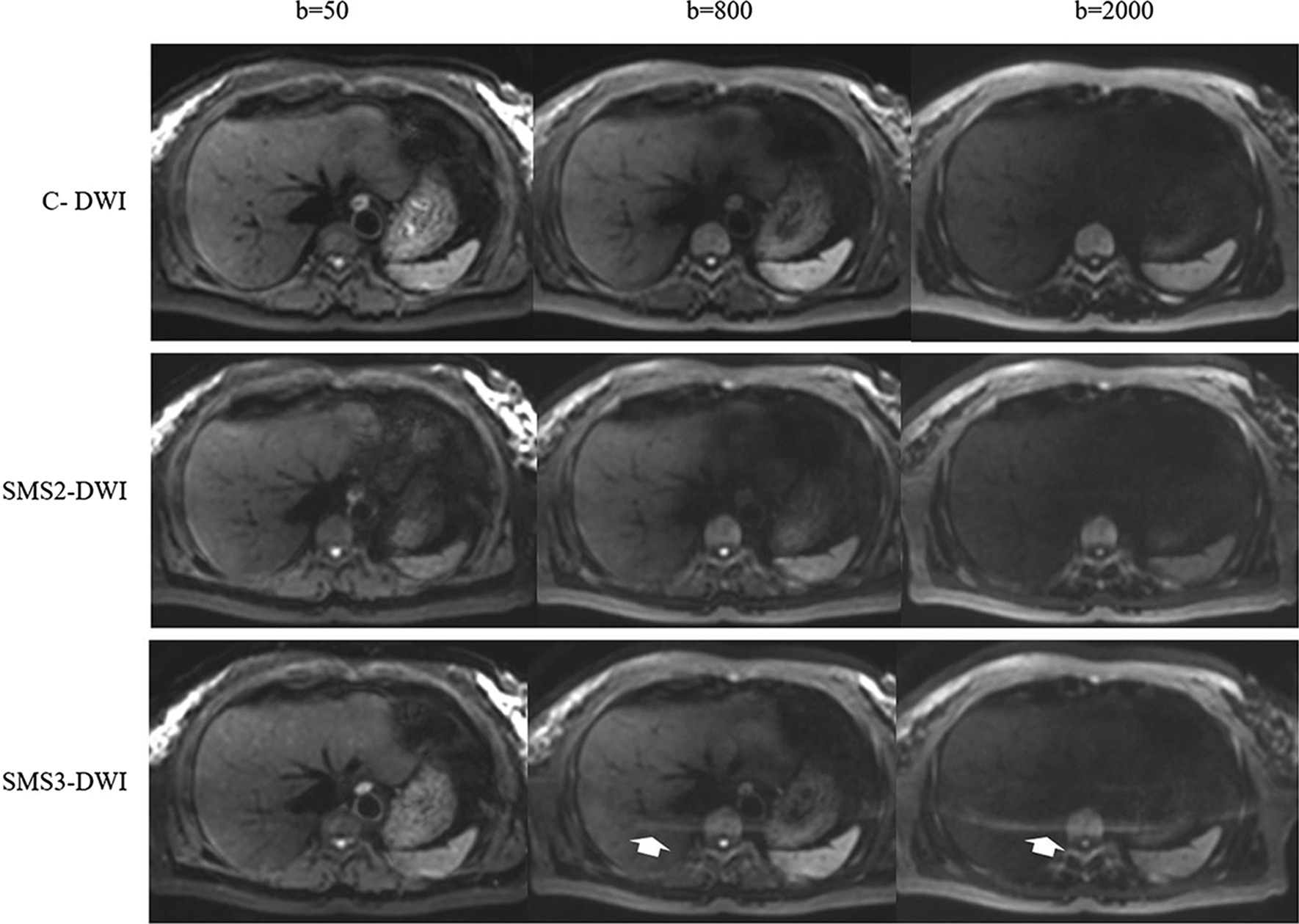
Comparisons of image quality of conventional DWI and SMS-DWI with different acceleration factors. Images were obtained in a 47-year-old healthy female volunteer with b values of 50, 800 and 2000 s/mm^2^ using SMS-DWI with acceleration factors of 2 (SMS2-DWI) and 3 (SMS3-DWI), and the conventional DWI sequence was used as a reference. The image quality of SMS2-DWI was rated as high as that of conventional DWI. In SMS3-DWI, substantially reduced image quality was found with signal loss in the central image portions as well as pronounced artefacts (white arrow), especially in the higher b-value images

**Fig. 2:**
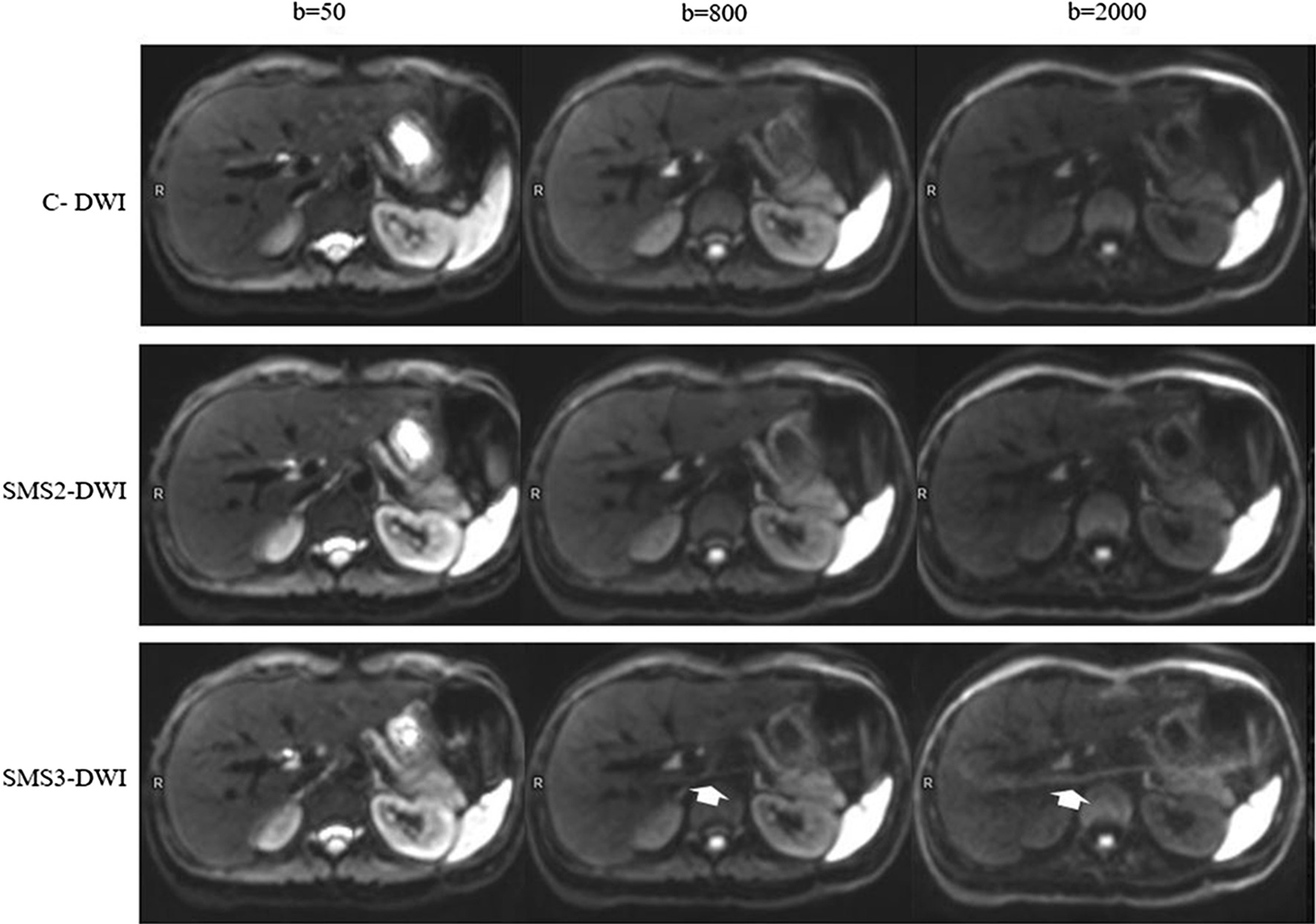
Comparisons of image quality of conventional DWI and SMS-DWI with different acceleration factors. Images were obtained in a 26-year-old healthy male volunteer with b values of 50, 800, and 2000 s/mm^2^ using SMS2-DWI, with acceleration factors of 2 (SMS2-DWI) and 3 (SMS3-DWI), and the conventional DWI sequence was used as a reference. The image quality of SMS2-DWI was rated as high as that of conventional DWI. In SMS3-DWI, substantially reduced image quality was found with signal loss in the central image portions as well as pronounced artefacts (white arrow), especially in the higher b-value images

Compared with C-DWI, SMS2-DWI significantly reduced the scan time by 51%, and compared with C-DWI, SMS3-DWI significantly reduced the scan time by 63%. Three b-value images of C-DWI, SMS2-DWI, and SMS3-DWI are shown in Figs. 1 and 2.

### Qualitative analysis of image quality

The interobserver agreement of the two readers was good for image quality assessment, with к values of 0.714 (95% confidence interval [CI], 0.634–0.795) for C-DWI, 0.639 (95% CI 0.554–0.725) for SMS2-DWI, and 0.653 (95% CI 0.579–0.726) for SMS3-DWI. The average image quality scores of the three sequences are shown in Table 2.

**Table 2:** Comparison of the results of qualitative image quality scores for the three sequences

Criteria	C-DWI	SMS2-DWI	SMS3-DWI	*P* values	*P* values	*P* values	*P* values
					C vs. SMS2	SMS2 vs. SMS3	C vs. SMS3
*Sharpness of the right lobe*							
b = 800	4.88 ± 0.40	4.84 ± 0.46	4.64 ± 0.60	< 0.001	1.000	0.144	0.066
b = 2000	4.16 ± 0.99	4.10 ± 1.00	3.74 ± 1.03	< 0.001	1.000	0.002	0.001
*Conspicuity of the left lobe*							
b = 800	4.63 ± 0.67	4.56 ± 0.71	4.40 ± 0.77	0.001	1.000	0.370	0.131
b = 2000	3.64 ± 1.13	3.45 ± 1.18	3.14 ± 1.05	< 0.001	0.268	0.008	< 0.001
*Visibility of intrahepatic vessels*							
b = 800	4.93 ± 0.26	4.70 ± 0.54	4.46 ± 0.71	< 0.001	0.190	0.109	< 0.001
b = 2000	3.88 ± 1.04	3.68 ± 1.11	3.20 ± 1.14	< 0.001	0.246	< 0.001	< 0.001
*Artefacts*							
b = 800	4.95 ± 0.22	4.69 ± 0.52	4.06 ± 0.75	< 0.001	0.207	< 0.001	< 0.001
b = 2000	4.70 ± 0.62	4.53 ± 0.64	3.49 ± 0.73	< 0.001	0.537	< 0.001	< 0.001
*Overall image quality*							
b = 800	4.80 ± 0.40	4.70 ± 0.49	4.13 ± 0.75	< 0.001	1.000	< 0.001	< 0.001
b = 2000	4.13 ± 0.82	4.05 ± 0.87	3.40 ± 0.69	< 0.001	1.000	< 0.001	< 0.001

Means and standard deviations of image quality obtained by two radiologists are summarized. b800, b = 800 s/mm^2^; b2000, b = 2000 s/mm^2^; C-DWI, conventional diffusion-weighted imaging; SMS2-DWI, simultaneous multislice accelerated DWI with an acceleration factor of 2; SMS3-DWI, simultaneous multislice accelerated DWI with an acceleration factor of 3

For all image quality parameters, there were no significant differences observed between C-DWI and SMS2-DWI in all b-value images (all *p* > 0.05) (Table 2). As shown in Figs. 1 and 2, compared with C-DWI, SMS2-DWI exhibited equivalent image quality and no artefacts.

There were no significant differences between SMS2-DWI and SMS3-DWI in the sharpness of the right lobe edge (*p* = 0.144), conspicuity of the left lobe (*p* = 0.370) or visibility of intrahepatic vessels (*p* = 0.109). SMS2-DWI exhibited better scores than SMS3-DWI for artefacts and overall image quality (all *p* < 0.01) in the images with a b-value of 800 s/mm^2^. For the images with a b-value of 2000 s/mm^2^, SMS2-DWI exhibited better scores than SMS3-DWI for all image quality parameters (all *p* < 0.01).

There were no significant differences between C-DWI and SMS3-DWI in the sharpness of the right lobe edge (*p* = 0.066) or conspicuity of the left lobe (*p* = 0.131). C-DWI exhibited better scores than SMS3-DWI for the visibility of intrahepatic vessels, artefacts, and overall image quality (all *p* < 0.01) in the images with a b-value of 800 s/mm^2^. For the images with a b-value of 2000 s/mm^2^, SMS2-DWI exhibited better scores than SMS3-DWI for all image quality parameters (all *p* < 0.05).

Artefacts of the liver could be observed in the high b-value (800 and 2000 s/mm^2^) SMS3-DWI images, and the artefacts became more obvious with increasing b-values (Figs. 1, 2).

### Quantitative analysis of signal-to-noise

The SNR values measured in the three DWI sequences are shown in Table 3. There were no statistically significant differences in the SNR between the three sequences in images with a b-value of 2000 s/mm^2^ (*p* = 0.110). For the images with a b-value of 800 s/mm^2^, there were no statistically significant differences between SMS2-DWI and C-DWI (*p* = 1.000) and no statistically significant differences between SMS2-DWI and SMS3-DWI (*p* = 0.059), whereas SMS3-DWI had a significantly lower SNR than C-DWI (*p* = 0.024).

**Table 3:** Results of the SNR analysis for the three sequences

	C-DWI	SMS2-DWI	SMS3-DWI	P values	P values	P values	P values
				Overall	C vs. SMS2	SMS2 vs. SMS3	C vs. SMS3
*SNR*							
b = 800	107.55 ± 48.56	103.51 ± 53.90	75.22 ± 57.60	0.015	1.000	0.059	0.024
b = 2000	50.88 ± 25.31	47.30 ± 23.11	38.95 ± 28.72	0.110	1.000	0.452	0.123

Means and standard deviations are shown for all sequences. SNR, signal-to-noise ratio; b800, b = 800 s/mm^2^; b2000, b = 2000 s/mm^2^; C-DWI, conventional diffusion-weighted imaging; SMS2-DWI, simultaneous multislice accelerated DWI with an acceleration factor of 2; SMS3-DWI, simultaneous multislice accelerated DWI with an acceleration factor of 3

### Quantitative analysis of DKI parameters and ADC values

**Fig. 3:**
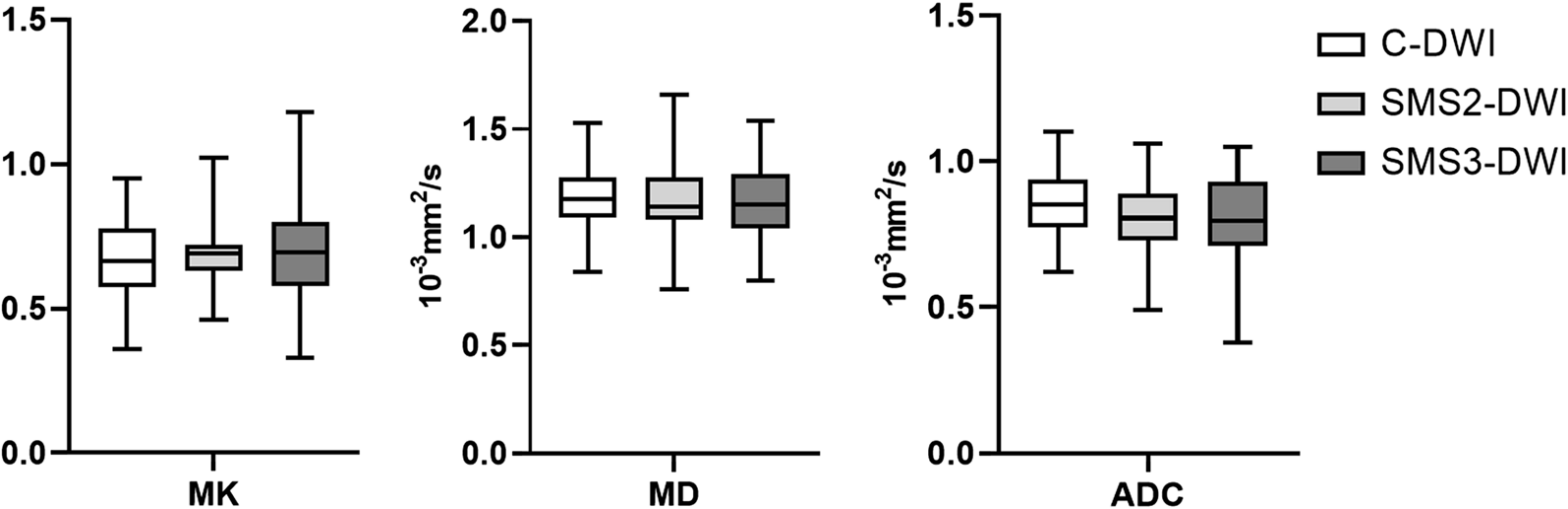
Box-and-whisker plots for MK, MD and ADC values of the liver in C-DWI, SMS2-DWI and SMS3-DWI sequences. The bottom and top of the boxes indicate the min and max of the values, respectively. The horizontal line inside the box indicates median values. The MK values of C-DWI, SMS2-DWI and SMS3-DWI were 0.67 ± 0.15, 0.68 ± 0.11, and 0.70 ± 0.18, respectively. The MD values of C-DWI, SMS2-DWI and SMS3-DWI were 1.19 ± 0.15 × 10^−3^ mm^2^/s, 1.17 ± 0.21 × 10^−3^ mm^2^/s, and 1.17 ± 0.18 × 10^−3^ mm^2^/s, respectively. The ADC values of C-DWI, SMS2-DWI and SMS3-DWI were 0.85 ± 1.12 × 10^−3^ mm^2^/s, 0.81 ± 1.24 × 10^−3^ mm^2^/s and 0.80 ± 1.56 × 10^−3^ mm^2^/s, respectively. There were no statistically significant differences between the three sequences (MK, *p* = 0.397; MD, *p* = 0.461; ADC, *p* = 1.346). With an increase in the acceleration factors, MK values showed an increasing trend and MD and ADC values showed a decreasing trend in the liver parenchyma

**Fig. 4:**
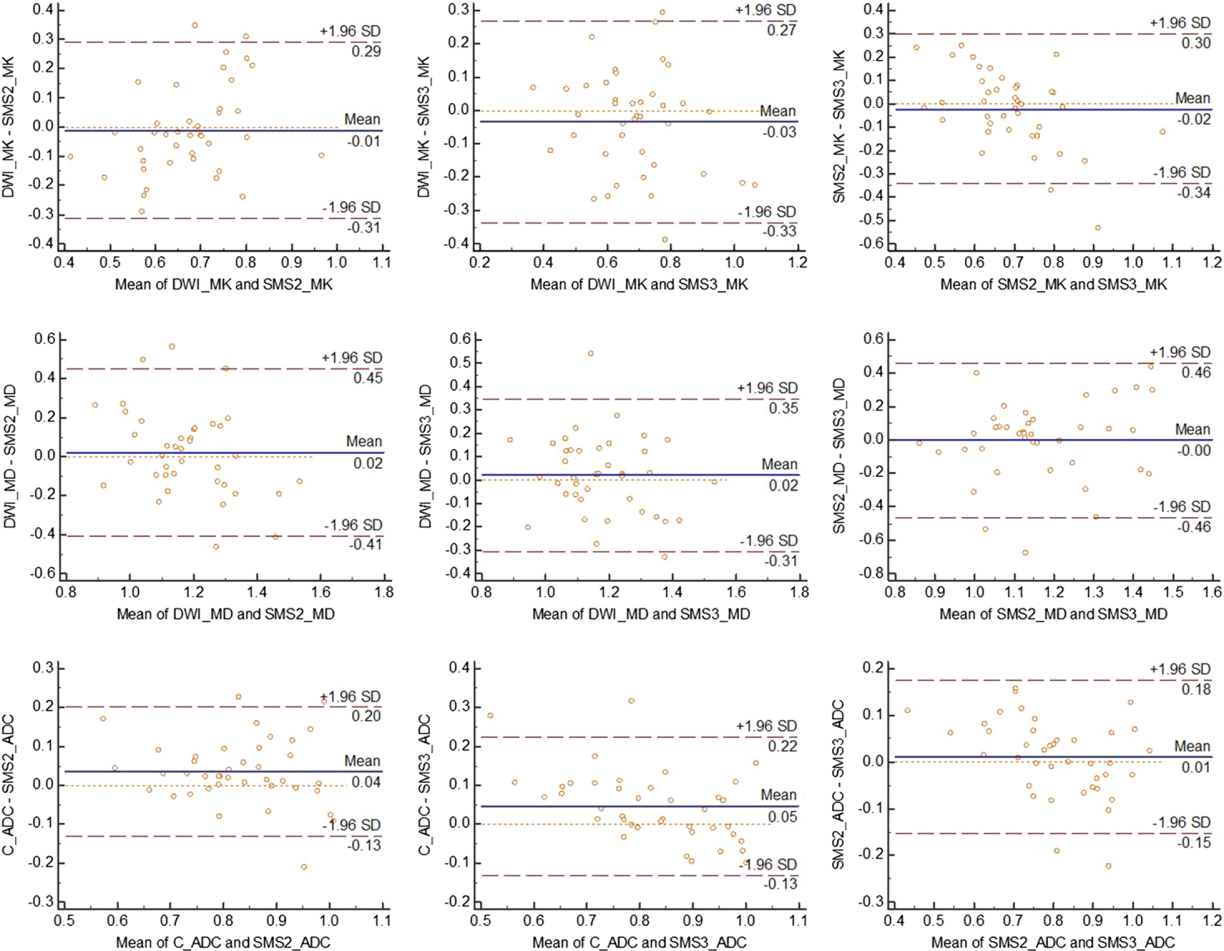
Bland–Altman plots comparing DKI parameters and ADC values between the three sequences. The first line represents MK values, the second line represents MD values, and the third line represents ADC values. The MD and ADC values are given in 10^−3^ mm^2^/s. The solid line indicates the mean absolute difference between two sequences, and the dashed lines indicate the 95% confidence interval of the mean difference. *SD* standard deviation

The MK values of C-DWI, SMS2-DWI and SMS3-DWI were 0.67 ± 0.15, 0.68 ± 0.11, and 0.70 ± 0.18, respectively. The MD values of C-DWI, SMS2-DWI and SMS3-DWI were 1.19 ± 0.15 × 10^−3^ mm^2^/s, 1.17 ± 0.21 × 10^−3^ mm^2^/s, and 1.17 ± 0.18 × 10^−3^ mm^2^/s, respectively. The ADC values of C-DWI, SMS2-DWI and SMS3-DWI were 0.85 ± 1.12 × 10^−3^ mm^2^/s, 0.81 ± 1.24 × 10^− 3^ mm^2^/s and 0.80 ± 1.56 × 10^−3^ mm^2^/s, respectively. There were no statistically significant differences between the three sequences (MK, *p* = 0.606; MD, *p* = 0.831; ADC, *p =* 0.264) (Fig. 3). Although there were no statistically significant differences in the DKI parameters between SMS2-DWI and C-DWI in this study, increased MK values and decreased MD values were observed with increased AFs in the liver. With an increase in the acceleration factor, the ADC values of the liver parenchyma showed a decreasing trend. The results from Bland–Altman plots are demonstrated in Fig. 4.

## Discussion

In the present study, SMS-DWI with an acceleration factor of 2 obviously decreased the scan time without a negative impact on the image quality and maintained comparable DKI parameters and ADC values compared with those of C-DWI. Although the scan time was significantly shortened for SMS3-DWI sequences, it had a negative impact on the image quality, especially at a high b-value of 2000 s/mm^2^.

The DKI model potentially reflects the non-Gaussian diffusion behaviour of water diffusivity in tissues using ultrahigh b-values above 1000 s/mm^2^ (1500–2000 s/mm^2^ in body imaging) [5, 6, 18]; thus, DKI could provide further information on tissue characteristics and has additional value in the prediction of microvascular invasion (MVI) of hepatocellular carcinoma (HCC) and in the assessment of post-therapeutic response in hypervascular HCC [3–5]. Obtaining ultrahigh b-value DWI images will induce a decreased SNR, increased distortion, susceptibility artefacts, and increased scan time [19].

In conventional diffusion acquisition, a single slice is excited, whereas in SMS-DWI, multiple slices are excited simultaneously, and the corresponding read-out contains these multiple slices. This permits the TR to be decreased, thus shortening the acquisition time, or allows more slices with thinner slice thicknesses to be scanned under the same TR [7, 9, 10]. Notably, the maximum number of simultaneous acquisition slices depends on the number of coil channels [7]. In theory, image acquisition in SMS-DWI can be accelerated to a large extent by increasing the applied acceleration factors. However, an arbitrary increase in the acceleration factors is expected to be associated with a decrease in image quality.

Our research indicates that SMS can shorten the scan time without reducing the image quality even in images with ultrahigh b-values of 2000 s/mm^2^ if an acceleration factor of 2 is used. Although there were no statistically significant differences in the DKI parameters between SMS-DWI and C-DWI in this study, SMS3-DWI should not be chosen in future studies due to the obviously decreased image quality and pronounced artefacts, especially in images with high b-values of 2000 s/mm^2^.

The MK values (unitless) of the DKI parameters reflect complex tissue microenvironments, such as tumour cells, necrosis, and inflammation. The MD values are the diffusion coefficients (unit: 10^−3^ mm^2^/s) related to Gaussian behaviour and similar to the ADC values that decrease with restricted diffusion. MK is determined by the SI decay curvature away from the plot that would be predicted by a monoexponential fit, whereas MD is determined by the slope of the SI decay plot as b approaches 0. Decreases in MD values and increases in MK values indicate abnormal diffusion behaviour in the organs. Although there were no statistically significant differences between the three sequences, with an increase in the acceleration factors, MK values showed an increasing trend and MD and ADC values showed a decreasing trend in the liver. This result could be due to a decrease in image quality that potentially affects the signal accuracies and influences the DKI parameter calculations in the SMS-DWI sequences.

Several studies have indicated that ADC values decrease when using SMS-DWI compared with C-DWI in the liver, pancreas and kidney [12, 15, 20]. One possible reason for the lower ADC values with higher acceleration factors is the shorter repetition time (TR) of the SMS sequence. In the present study, the TR was 5200, 2400, and 1800 ms for C-DWI, SMS2-DWI, and SMS3-DWI, respectively. The lower TR may lead to a reduced signal due to T1 saturation effects, particularly with the high b-value images that had higher AFs, resulting in decreased ADC values.

Obele et al., who investigated the SMS technique with an acceleration factor of 2 in free breathing, noted that there were slightly lower ADC values compared with those of a conventional DWI sequence without statistical significance in the liver [12]. Another reason may be that we scanned the 3 b-values together, which induced an increase in TE and led to a lower SNR in the 2000 s/mm^2^ b-value images, which also influenced the ADC values to some degree. When the ADC value was used for diagnosis in the liver, there were possibly lower ADC values in SMS sequences. Considering scan time, image quality and ADC values, an acceleration factor of 2 would be the best option for a clinical workflow in the liver.

There are several limitations of the study. First, the SMS-DWI and C-DWI sequences were scanned during free breathing and without respiratory-triggered techniques in our study. Respiratory motion and hepatic local motion may impact the image quality and challenge the reliability of the computation of DKI parameters to some degree. Several studies have indicated that compared with conventional DWI, SMS-DWI combined with respiratory triggering could improve image quality in the liver and kidney [21–23]. SMS-DWI sequences with respiratory compensation techniques should be implemented in the future.

Second, a comparison and assessment of DKI parameters using SMS-DWI were performed in relatively young and healthy participants. The diffusion coefficients can differ in normal tissues, diseased tissues, and focal lesions. Thus, the DKI parameters based on SMS-DWI for evaluating diffusive diseases, such as liver fibrosis, cirrhosis, and focal lesions, need to be further studied.

## Conclusion

In conclusion, compared with conventional DWI sequences, SMS-DWI using an acceleration factor of 2 could reduce the scan time significantly and without negative effects on image quality and comparable DKI parameters and ADC values. Based on the present study results, SMS-DWI can promote DKI sequences in clinical settings. Further studies are needed to investigate the clinical diagnostic performance of DKI based on the SMS technique for evaluating diffuse diseases and focal lesions of the liver.

## Data Availability

The datasets used and analysed in the current study are available from the corresponding author on reasonable request

## Abbreviations

DKI: Diffusion kurtosis imaging
SMS: Simultaneous multislice
MK: Apparent mean kurtosis
MD: Apparent mean diffusivity
ADC: Apparent diffusion coefficient
SNR: Signal-to-noise ratio
ROI: Region of interest
